# Injection of mRNA isolated from trophozoites of *Giardia intestinalis* induces expression of three types of chloride currents in *Xenopus laevis* oocytes.

**DOI:** 10.14814/phy2.14029

**Published:** 2019-06-12

**Authors:** Arturo Ponce, Alejandro Ogazon del Toro, Lidia Jimenez, Leticia Eligio‐Garcia, Enedina Jimenez‐Cardoso

**Affiliations:** ^1^ Department of Physiology Biophysics and Neurosciences. Center for Research and Advanced Studies Mexico City Mexico; ^2^ Department of Physiology Biophysics and Neurosciences. Center for Research and Advanced Studies Mexico City Mexico; ^3^ Department of Physiology Biophysics and Neurosciences. Center for Research and Advanced Studies Mexico City Mexico; ^4^ Parasitology Research Laboratory Children Hospital of México “Federico Gomez” Mexico City México; ^5^ Parasitology Research Laboratory Children Hospital of México “Federico Gomez” Mexico City México

**Keywords:** Chloride channels, electrophysiology, *Giardia Intestinalis*, *Xenopus laevis*

## Abstract

*Giardia lamblia* is one of the most important worldwide causes of intestinal infections, yet little is known about its cellular physiology, especially the diversity of ionic channels that this parasite expresses. In this work, we show that injection of mRNA isolated from trophozoites of *Giardia*, into *Xenopus laevis* oocytes, induces expression of three types of chloride currents (here referred to as ICl‐G1, ICl‐G2, and ICl‐G3), which have different biophysical and pharmacological properties. ICl‐G1 currents show inward rectification and voltage dependence are enhanced by hypotonicity, show a selectivity sequence of (I > Br > Cl > F), and are inhibited by NPPB, DIDS, SITS, 9AC, DPC, and Zinc. These findings suggest that ICl‐G1 is the result of expression of chloride channels related to ClC2. ICl‐G2 currents show outward rectification and are dependent of intracellular calcium, its selectivity sequence is (Cl > Br > I > F) and are inhibited by NPPB, DIDS, SITS, 9AC, DPC, niflumic acid, tannic acid, and benzbromarone. These findings suggest that they are produced by calcium dependent chloride channels (CaCC). The third type of currents (ICl‐G3) appears only after a hypoosmotic challenge, and has similar properties to those described for ICl‐swell, such as outward rectification, instant activation, and slow inactivation at large depolarizing voltages. They were blocked by NPPB, DIDS, 9AC, NIf, DCPIB, and tamoxifen. Our results indicate that *Giardia intestinalis* has at least three types of anion conductances.

## Introduction

*Giardia intestinalis,* also known as *Giardia lamblia*, is a flagellated protozoan of great importance because it is the cause of giardiasis, one of the most frequent enteroparasitic infections, whose most common symptoms are diarrhea, abdominal pain, vomiting, and weight loss. Giardiasis is spread worldwide, affecting around 280 million people each year, mostly from developing countries. Children under the age of 5 years old are more vulnerable to acquire this disease. *Giardia* is a noninvasive, flagellated, microaerophilic protozoan that resides and multiplies by binary division on the surface of the first portions of the small intestine, at a slightly alkaline pH that favors its development. It has a simple life cycle, consisting of two stages: A free dwelling, proliferating trophozoite, and an infecting cyst. Despite its importance as a health problem, relatively few is known about the cellular and molecular physiology of *Giardia* (Adam 1991; Gillin et al. 1996).

Ion channels are an essential component of the membrane of living cells. Its basic function is to regulate, selectively, the transmembrane flux of ions of physiological relevance, such as Na^+^, K^+^, Ca^++^_,_ and Cl^−^ (Hille 2001). Although they are widely diverse, both in structure and function, ion channels are most generally classified according to the type of ion for which they are more selective (Alexander et al. 2011). Among the distinct types of ion channels found in nature, chloride channels are of striking relevance. Chloride channels have been found to be expressed in all types of cells, both in prokaryotes and in eukaryotes, participating in a wide variety of physiological processes, including excitability, cell proliferation, motility, cell volume regulation, and cellular recognition of their environment, among many others (Nilius and Droogmans 2003). So far, several types of chloride channels have been identified, either by its molecular or its functional properties, including voltage‐dependent chloride channels (ClC),cystic fibrosis transmembrane regulator (CFTR), calcium‐activated chloride channels (CaCC), and volume‐regulated chloride channels (VRAC) (Poroca et al. 2017; Jentsch 1996; Grinstein et al. 1982). Although they have been described in almost all types of cells and tissues, there are only a few reports describing chloride channels in protozoans (Delgadillo et al. 2002; Salas‐Casas et al. 2006; Moreno‐Galindo et al. 2014).

In a previous work, we resorted to an indirect experimental approach to study the biophysical and pharmacological properties of potassium currents of trophozoites of *Giardia* (Ponce et al. 2013). It consists of the injection of mRNA isolated from trophozoites in oocytes of *Xenopus laevis*, a South African clawed frog. Because it has been found that, in most cases, oocytes synthesize exogenous proteins after mRNA injection, this method has been used to describe the biophysical and pharmacological properties of ion channels and receptors from diverse species, including protozoan parasites like *Entamoeba histolytica* (Delgadillo et al. 2002; Salas‐Casas et al. 2006) and *Leishmania* (Figarella et al. 2007; Lagos et al. 2007).

In the present work, we used the same experimental approach to study the properties of chloride currents from *Giardia intestinalis*. We show here that injection of mRNA from trophozoites induces anion selective currents and describe their biophysical and pharmaceutical properties.

## Materials and Methods

### Production and purification of mRNA from *Giardia*

Total RNA was extracted and purified from trophozoites of *Giardia*, strain Portland‐1 (ATCC‐30888), grown axenically in TYI‐S‐33 media, as described by Farthing et al. (1983). The extraction of total mRNA was made with the Ambion^®^ RNAqueous^®^ Kit (Cat. Number AM1912, Life Technologies, Carlsbad, CA). Messenger RNA was purified with the Dynabeads mRNA Purification Kit (Cat 61006, Ambion) following the procedures described by the manufacturer. Cleaved mRNA, which was made for control purposes, was produced by incubating mRNA with 1 *μ*g/*μ*L RNase A (12091‐039, Invitrogen) at 37°C for 30 min.

### Dissection of oocytes and injection

General procedures were as described elsewhere (Ponce et al. 2013). In order to harvest oocytes, female frogs were anesthetized by immersion in a tank containing 0.1% Tricaine (MS‐222, Sigma‐Aldrich A‐5040, St. Louis, MO). Ovarian lobes were partially removed and immersed in a Petri dish containing saline solution OR2 (see below for composition) and kept at 18°C for 8 h in a low‐temperature incubator (VWR 2005). Oocytes were manually detached from the ovarian lobes. To detach the follicular layer, oocytes were incubated with collagenase (type 1 (C9891, Sigma, 2 mg/mL) in calcium‐free ND96 solution with gentle agitation at room temperature. Then, oocytes were rinsed twice with ND96 solution and kept overnight at 18°C. Stage IV oocytes were selected for injection of mRNA. Injection was performed with a micropipette (Manual Oocyte Microinjection Pipet, cat No. 3‐000‐510‐X, Drummond Scientific Company, Broomall, PA), which was held on a mechanic micromanipulator (MN153, Narishige Scientific Instruments, Tokyo, Japan). A stereoscopic microscope (SMZ‐1500, Nikon Corporation, Shinjuku, Tokyo, Japan) was used to monitor the injection process. Each oocyte was injected with 50 nL of degraded or intact mRNA (1 *μ*g/*μ*L). After injection, oocytes were incubated at 16°C in ND96 supplemented with gentamicin (5 mg/mL) until recording of ion currents.

The trial was reviewed and approved by the Internal Committee of the Center for Research and Advanced Studies (CICUAL) in accordance with the Mexican Official Normativity for Ethical Care and Management of laboratory animals (NOM‐062‐ZOO).

### Electrophysiology

Ion currents were recorded from oocytes, using the standard two‐electrode voltage clamp technique (Stühmer 1992; Schwarz and Rettinger 2003). The setup for electrophysiology consisted of a TEV 200 amplifier (Dagan Corporation, Minneapolis, MN) connected to a PC computer through an analog–digital converter (Digidata 1322A, Molecular Devices LLC, Sunnyvale, CA). Voltage clamp‐stimulating protocols and acquisition of currents were made with the Clampex module of the software suite pclamp 6.0 (Molecular Devices LLC). Microelectrodes, with a tip resistance of 1–2 mΩ, were made by pulling glass tubing (GT‐15, Warner Instruments, Hamden, CT) on a Brown–Flaming type puller (P87, Sutter Instruments, Novato, CA) and filled with KCl 3 mol/L. Reference electrodes were connected to a recording chamber through glass bridges filled with 200 mmol/L NaCl in 2% agarose (A9539, Sigma‐Aldrich). For recording of ion currents, oocytes were placed in a chamber filled with saline recording solution (whose composition is described below) and impaled with both electrodes. This procedure was verified, optically and electrically, by monitoring a sudden change in the membrane potential. The external bathing solution was continuously perfused by gravity at a rate of 5 mL/min. Distinct protocols of voltage were used as detailed below (see Results). Current signals were processed online with a low‐pass filter, at a cutoff frequency of 200 Hz, and sampled at 34.5 Hz. All assays were performed at room temperature.

### Solutions

Table 1 shows the composition of the distinct saline solutions used in this work. OR2 was used for dissection procedures. Oocytes were kept in ND96 while injecting; and incubated in ND96C. SRBS was used as a starting recording bath solution, it has the same composition as ND96, but 36 mmol/L NaCl was replaced by TEA‐Cl, in order to exclude potassium currents. We used two solutions for hypoosmotic challenge assays; MRBS‐ISO in which we replaced 36 mmol/L NaCl by 72 mmol/L mannitol (in order to keep osmolarity as in SRBS) and MRBS‐HO (same saline composition as MRBS‐ISO, without mannitol). For selectivity assays, solutions were made with the same cationic composition as SRBS but were prepared from hydroxides (NaOH, KOH, Ca(OH)_2_, Mg(OH)_2_, TEA‐OH) instead of chloride salts (NaCl, KCl, CaCl_2_, MgCl_2_, TEA‐Cl) and titrated to pH7.4 with acid from the testing anion (HBr, HI, HSCN, HF, or MSA).

**Table 1:** Composition of solutions

Solution name	NaCl	KCl	CaCl_2_	MgCl_2_	Na_2_HPO4	HEPES	TEA‐Cl	Na‐pyruvate	Gentamicin	Mannitol
OR2	82.5	2.5	1	1	1	5	0	0	0	0
ND96	96	2	1	1	0	5	0	0	0	0
ND96‐C	96	2	1	1	0	5	0	6	100 *μ*g/mL	0
SRBS	60	2	1	1	0	5	36	0	0	0
MRBS‐ISO	24	2	1	1	0	5	36	0	0	72
MRBS‐HO	24	2	1	1	0	5	36	0	0	0

### Chemicals and drugs

The following drugs and chemicals, used in this work, were all purchased from Sigma‐Aldrich:

NPPB (5‐nitro‐2‐(3‐phenylpropylamino)benzoic acid), CAS #: 107254‐86‐4;DIDS (4,4′‐Diisothiocyanatostilbene‐2,2′‐disulfonic acid disodium salt hydrate), CAS #: 207233‐90‐7;SITS (4‐Acetamido‐4′‐isothiocyanato‐2,2′‐stilbenedisulfonic acid disodium salt hydrate), CAS #:51023‐76‐8:9AC (9‐anthracenecarboxylic acid), CAS #: 723‐62‐6):DPC(Diphenylamine‐2‐carboxylic acid), CAS #:91‐40‐7;Zinc, CAS #:7440‐66‐6;Niflumic acid, CAS #:4394‐00‐7;Tannic acid, CAS #:1401‐55‐4;Benzbromarone, CAS #:3562‐84‐3;DCPIB(4‐(2‐butyl‐6,7‐dichloro‐2‐cyclopentylindan‐1‐on‐5‐yl)oxybutyric acid), CAS #: 82749‐70‐0;Tamoxifen ((Z)‐1‐(p‐Dimethylaminoethoxyphenyl)‐1,2‐diphenyl‐1‐butene, trans‐2‐[4‐(1,2‐Diphenyl‐1‐butenyl)phenoxy]‐N,N‐dimethylethylamine), CAS #: 10540‐29‐1; BAPTA(1,2‐Bis(2‐Aminophenoxy)ethane‐N,N,N′,N′‐tetraacetic acid), CAS #: 85233‐19‐8; ionomycin, CAS #: 56092‐81‐0.

### Analysis of data

Calculation of relative permeabilities (*P*_*x*_/PCl) was made with the Goldman–Hodgkin–Katz equation (Hille 2001):

$$\frac{P_{x}}{P_{\mathrm{Cl}}}=\frac{\left(\left[{\text{Cl}}_{O}\right]\;e\frac{\Delta \text{EF}}{\text{RT}}\right)-\left[{\text{Cl}}_{\mathrm{res}}\right]}{\left[x_{O}\right]}$$

Where Δ*E* is the shift in reversal potential, [Cl_o_] is the extracellular concentration of chloride in the bathing media, [xo] is the extracellular anion concentration in anion‐substituted ND‐96, and [Clres] is the remaining Cl^−^ concentration in the anion‐substituted media.

All signal processing procedures, such as filtering and compensation of linear components (capacitive and leak currents), as well as measurement of ion current magnitude were made offline with the clampfit module of the Pclamp software suite (Molecular Devices). Mathematical procedures such as the transformation of data and curve fitting were made with Sigmaplot 12 (Systat Software, Bangalore, Karnataka, India). Statistical calculations and tests were made with Microsoft Excel (Office 2016). Results are reported as a mean ± standard error (number of cases). Differences among treatment groups were analyzed using Student's *t*‐test, assuming equal variances, with Null difference among groups. Multiple comparisons were made with one‐way analysis of variance (ANOVA), followed by Student's *t*‐test corrected for multiple comparisons (Fisher's exact test). *P* < 0.05 was taken as a minimal criterion to consider Null hypothesis rejection.

## Results

### Injection of mRNA, isolated from *Giardia*, induces expression of anion currents in *Xenopus oocytes*

**Figure 1:**
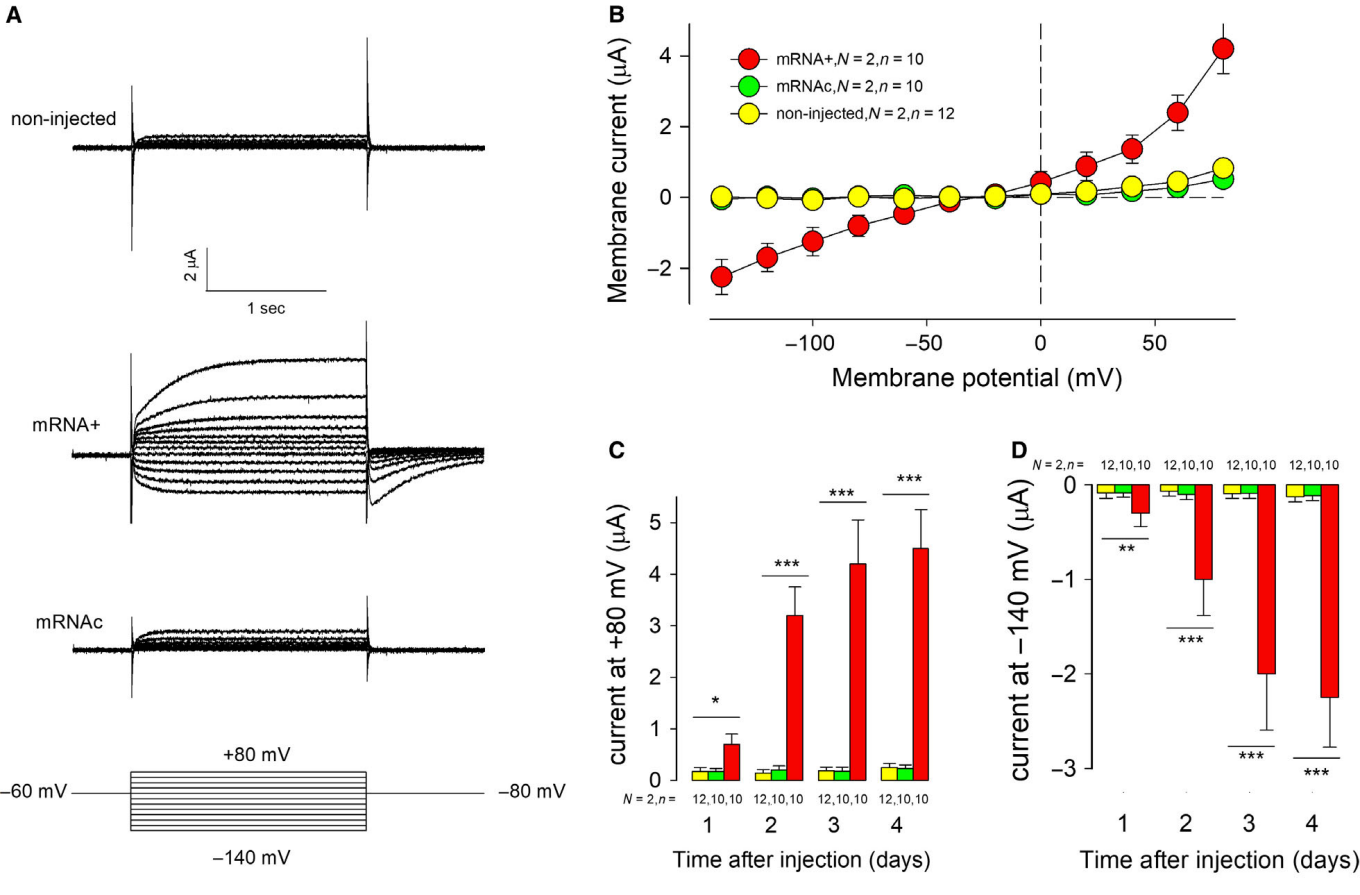
Injection of mRNA, isolated from cultured trophozoites of *Giardia intestinalis*, into *Xenopus laevis* oocytes, induces expression of exogenous chloride currents. (A) Representative series of currents obtained, in response to a voltage clamp protocol, shown in the lowest part, from oocytes that were either noninjected, injected with mRNA of *Giardia*, or cleaved mRNA. (B) Relationship between the average amplitude of current and the testing membrane voltage of each of the three experimental conditions (C–D) Time course of expression of the outward component of current after injection of *Giardia* mRNA. Bars show the average (±SE) magnitude of current at +80 mV (C) or at −140 (D) obtained from noninjected oocytes (yellow), or injected with cleaved mRNA (green) or intact mRNA (red). One‐way ANOVA , run on each group, indicates significant differences for intact mRNA‐injected oocytes (*P* < 0.01).

Initially, we compared the biophysical properties of anion currents from noninjected oocytes, with those from oocytes that had been injected with mRNA, as well as with oocytes injected with degraded mRNA. The average resting membrane potential of oocytes injected with intact mRNA (38.2 ± 0.6 mV, *N* = 2, *n* = 12) was not statistically different (ANOVA, *P* = 0.11) from that of noninjected (40.2 ± 0.7 mV, *N* = 2, *n* = 12), nor from those injected with degraded mRNA (38.7 ± 0.7 mV, *N* = 2, *n* = 12). Figure 1A shows three representative series of ionic currents obtained from oocytes that were either noninjected (upper), or injected with intact mRNA intact (middle) or degraded mRNA (lower). All of them were recorded 3 days after injections, as a response to a stimulation protocol (shown in the lowest part), consisting of a series of testing square voltage pulses, ranging from −140 mV to 80 mV in steps of 20 mV. In between each test pulse the voltage was clamped at −80 mV for 5 sec, then changed to −60 mV for 0.5 sec. All series of currents are shown on the same scale of time and amplitude of the current. Oocytes were bathed in SRBS, containing 36 mmol/L TEA‐Cl to exclude potassium currents. It is shown that oocytes injected with intact mRNA isolated from *Giardia*, express ion currents (ICl‐G), whose magnitude and kinetics differ clearly from those recorded from noninjected oocytes or from oocytes injected with degraded mRNA. These results indicate that such currents are expressed, as a result of injection of mRNA from *Giardia*. ICl‐G currents activate gradually until reaching a steady amplitude, both in the hyperpolarizing and depolarizing range of voltage, although the rate of activation in the inwardly range is faster (tau = 103 ± 7 msec, *n* = 5) than in the outwardly (tau = 323.2 ± 12 msec, *n* = 5), suggesting that there are two types of currents involved. Figure 1B shows the relationship (IV) between the average steady amplitude of current versus the testing voltage, which ranged from −140 to +80 mV. It can be observed that the IV relationship of currents obtained from oocytes injected with mRNA is noticeably distinct from those obtained from noninjected oocytes or from those injected with cleaved mRNA, both in the hyperpolarizing and depolarizing range. Figure 1C and D show the time course (days after injection) of the average magnitude of currents at +80 mV and at −140 mV, respectively, from the three experimental groups. From the first day after injections, the average magnitude of the current of oocytes injected with intact mRNA is significantly distinct from the corresponding values from oocytes noninjected or injected with cleaved mRNA. In both cases (1C and 1D), the average magnitude of the current of oocytes injected with intact mRNA increases progressively over time, suggesting that it is due to a gradual synthesis and recruitment of exogenous ion channels to the oocyte′s plasma membrane.

### The inwardly rectifying component of current (ICl‐G1) is activated by hyperpolarizing conditioning voltage

**Figure 2:**
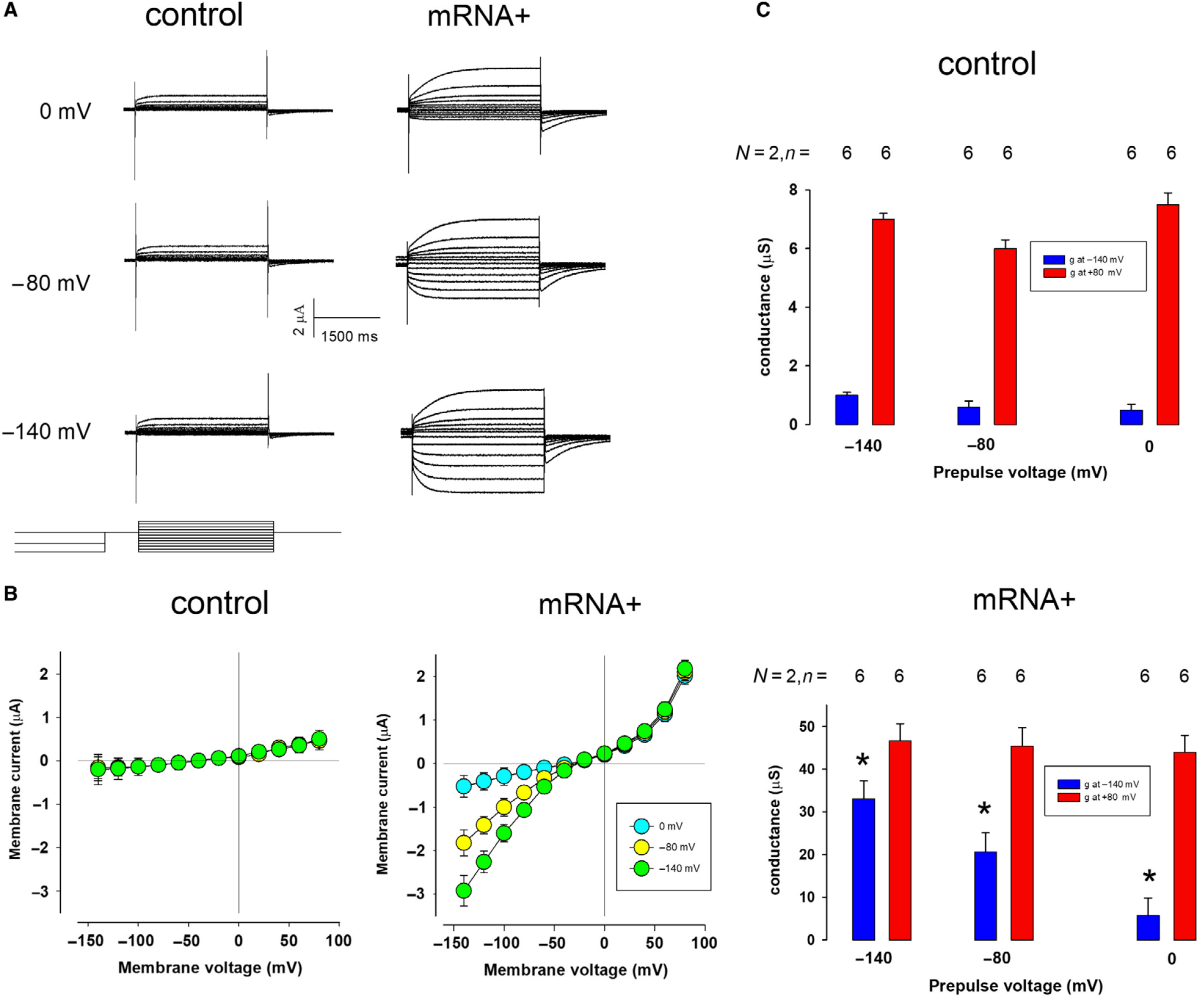
Hyperpolarizing conditioning voltage enhances the inwards component of current (ICl‐G1). (A). Representative series of currents obtained, from the same oocyte, either control (left column) or injected with mRNA (right column), in response a voltage clamp protocol (depicted in the lower part of left column), consisting of step pulses ranging from −140 to + 80 mV in steps of 20 mV. Each testing pulse was preceded by a prepulse of 0, −80, or −140 mV, during 5 sec followed by an interpulse of 0 mV during 0.2 sec. In the upper row a prepulse of 0 mV was given; In the middle one a prepulse of −80 mV, and in the lower it was −140 mV. (B) *I*–*V* relationship of the three conditions described, from control (left) and mRNA‐injected oocytes. The latter shows a change of conductance in the inwards part, but not in the outwards one. (C) Comparison of the average slope conductance at −140 and + 80 mV, from control (upper) and mRNA‐injected (lower) oocytes obtained from recordings with a prepulse of either −140, −80, or 0 mV. One‐way ANOVA indicates statistically significant differences for conductance of mRNA‐injected oocytes at −140 (*P* < 0.01) but not at +80 mV. The same statistical analysis indicates no statistically significant difference for conductances of control oocytes.

The difference in both the kinetics of activation kinetics and the conductance in the hyperpolarizing and depolarizing voltage range, shown above, prompted us to consider the possibility that two types of currents are actually being expressed. Therefore, we made experimental assays to distinguish both types of currents. Firstly, we evaluated whether any or both of these currents depend on the transmembrane voltage. Figure 2A shows representative series of ion currents, obtained from the same oocyte, either control (left column) or injected with mRNA (right column) with the same protocol of test voltage pulses, except that, a conditioning prepulse of variable voltage (0, −80 or −140 mV), lasting 5 sec, was applied before each testing pulse. This procedure does not produce notorious changes in the currents of control oocytes, but in those from injected with mRNA, the magnitude of inward currents increases significantly when the membrane is subjected to hyperpolarizing conditioning pulses, whereas outward currents do not undergo a notorious change. Figure 2B compares the relationship between the testing voltage and the mean value of the current (±SE) of all three prepulse voltage settings, from control (left) and mRNA‐injected (right) oocytes, whereas Figure 2C, that compares the slope conductance at −140 and +80 mV for each of the prepulse voltages tested (0, −80, and −140 mV). Statistical analysis (one‐way ANOVA) indicates that in values obtained from control oocytes, there is no statistical difference, neither at −140 nor at +80 mV. In contrast the equivalent values were statistically different at ‐140 mV (*P* < 0.01) but not at +80 mV.

Therefore, these results support our hypothesis that there are two distinct types of chloride currents expressed in oocytes after injection of *Giardia* mRNA. One of them shows inward rectification and depends on transmembrane voltage. It is worth to observe that these currents, which we will refer to now as ICl‐G1, have biophysical properties similar to those described for ClC2, a type of channel that belongs to ClC, a family of voltage‐dependent chloride channels (Stölting et al. 2014).

### The outwardly rectifying component of current (ICl‐G2) depends on intracellular calcium

**Figure 3:**
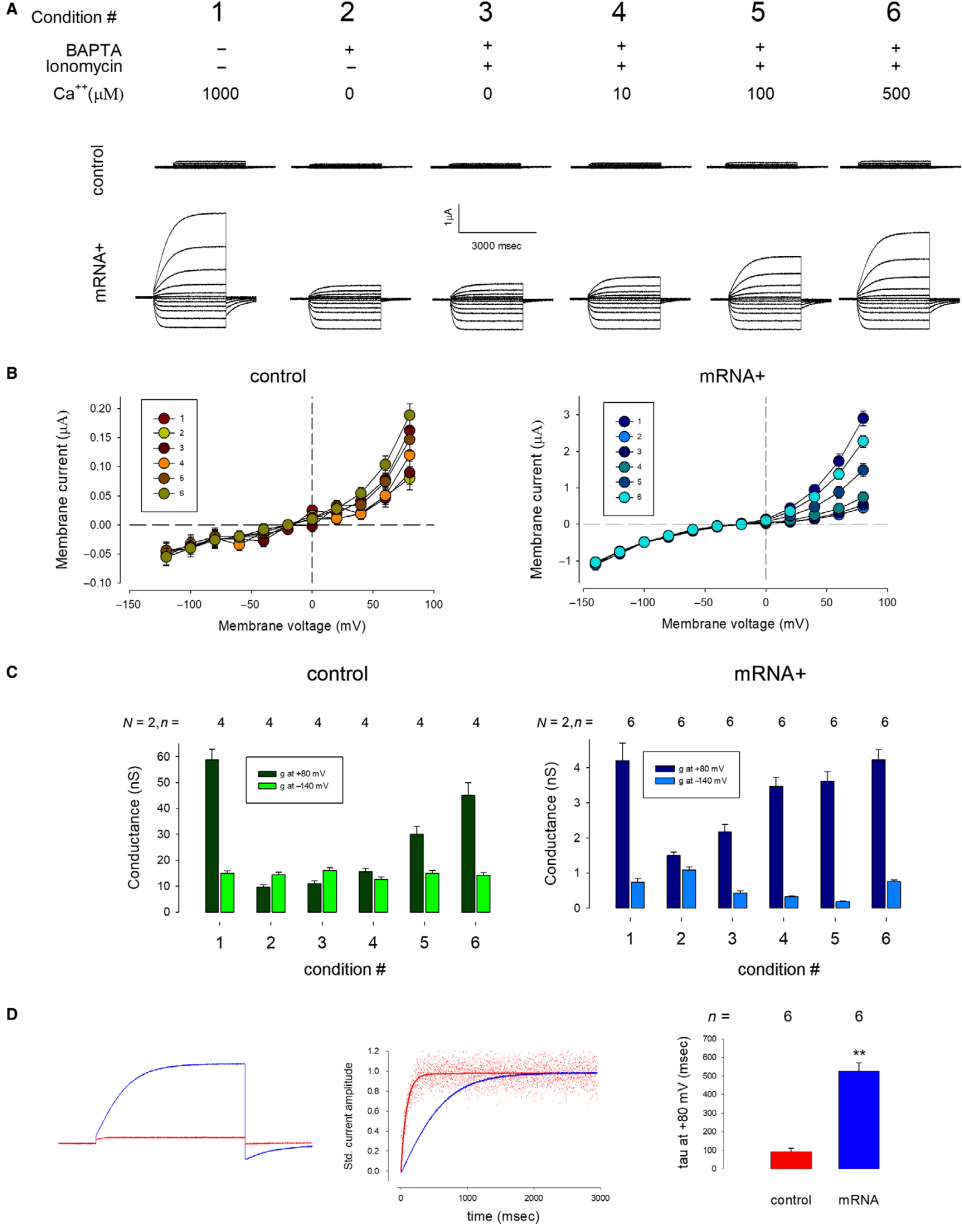
The outward component of current (ICl‐G2) depends of the intracellular calcium concentration. (A) Representative series of currents from control (upper row) and mRNA‐injected (lower row) oocytes, obtained under the experimental conditions described in the upper part. (B) *I–V* relationship of control (left) and mRNA‐injected (right) oocytes treated with the experimental conditions described in the upper part of figure 5. (C) Comparison of the average slope conductance at −140 (blue) and +80 mV (red) of control (left) and mRNA‐injected oocytes. one‐way ANOVA within groups indicates statistically significant differences for treatments at −140 mV (*P* < 0.01) but not for treatments at +80 mV of both control and injected mRNA. (D) (left) Comparison of the magnitude and kinetics of currents obtained in response to a test pulse of + 80 mV of control (red) and mRNA‐injected (blue) oocytes. (middle) Currents are shown standardized to compare the kinetics of activation. (right) Statistical comparison of the average value of the time constant (tau), obtained from fitting the standardized currents at +80 mV of control (red) and mRNA‐injected currents, indicates a significant difference (*P* < 0.01).

To determine if ICl‐G depends of intracellular calcium, we made recordings under six different experimental conditions (as described in the uppermost part of Fig. 3A). Figure 3A shows two rows of representative series of currents obtained from a control (upper) and a mRNA‐injected (lower) oocyte. All those series were produced with the same protocol of stimulation described in Figure 1. The first pair (condition 1) compares currents obtained from a control and an injected oocyte while bathed in SBRS. The next pair (condition 2) shows currents, from the same oocytes, 5 min after injection of 50 nL of BAPTA 100 mmol/L, to chelate intracellular calcium, (as described by Hartzell 1996) and recorded with SRBS with no calcium. This procedure notoriously reduced the magnitude of currents in the depolarizing but not in the hyperpolarizing range of voltage, both in control as in injected oocytes, as it can be observed in Figure 3B, that shows the IV relationships of control (left) and mRNA‐injected (right) and Figure 3C, that compares the slope conductance at −140 and +80 mV of control (left) and mRNA‐injected oocytes. Next, to allow the influx of calcium from the bath media, we incubated BAPTA‐injected oocytes with ionomycin 1 *μ*mol/L (as described by Yoshida 1992), and made recordings of currents after perfusing the external media with solutions containing an incrementing concentration of calcium (0, 10, 100, and 500 *μ*mol/L), the result is shown in Figure 3A (conditions 3–6) and in Figure 3B and C. It can be observed that the addition of calcium in the extracellular medium increases the magnitude of currents in the depolarizing but not in the hyperpolarizing range, both in control as in injected oocytes. Although both control and mRNA‐injected oocytes responded in the same way to treatments, the magnitude of currents is clearly distinct. The mean slope conductance at +80 mV of injected oocytes (59 ± 5 nS) is significantly (*P* < 0.01) greater than that of control oocytes (5.4 ± 0.9 nS). Besides the notorious difference in the magnitude of currents, the kinetics is clearly distinct. Figure 3D compares the kinetics of currents (at +80 mV) of a control (red) and mRNA‐injected (blue), as recorded (left) and standardized (middle). Also shown (right) that fitting of the standardized current (at +80 mV) to the exponential equation:

f=y0+a∗(1−exp(−x/tau))

Indicates a tau of 526.5 ± 43 msec (*n* = 6) of currents obtained from mRNA‐injected oocytes, which is significantly larger (*P* < 0.005) than the tau of currents obtained from control oocytes (92 ± 18 msec, *n* = 6).

### ICl‐G1 and ICl‐G2 have a distinct sequence of selectivity

**Figure 4:**
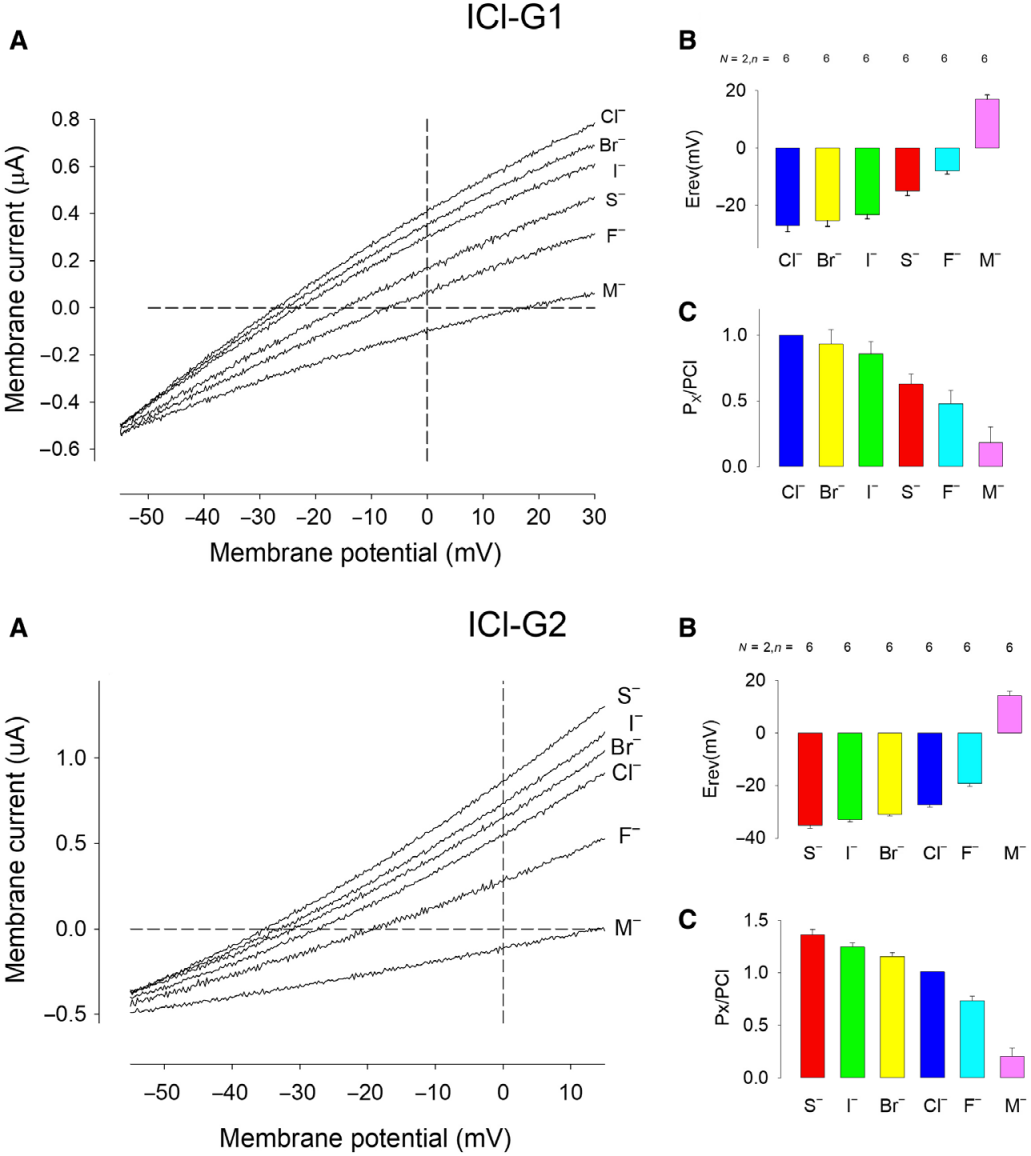
ICl‐G1 and ICl‐G2 have different selectivity sequences. (A) representative traces of current obtained in response to ramp voltage protocol, while the oocyte was bathed with a saline solution containing either of six distinct anions: Methanesulfonate‐(M), I‐,Br‐,Cl‐,F‐, or Thiocyanate‐(S). (B) Average value of the reversal potential from eight oocytes from two frogs. (C) Average relative permeability values of the distinct anions.

To determine the selectivity profile of the two types of current described above, we made recordings of ion currents in response to ramp functions, under suitable conditions to reveal either one of them: To disclose ICl‐G1, we used oocytes previously injected with BAPTA, whereas to disclose ICl‐G2, we stimulated oocytes at 0 mV before recordings. In both cases, we made ramp testing pulses, from −60 to + 30 mV. The recording of currents was made from oocytes immersed in a bath solution in which we substituted 96 mmol/L chloride with either of the testing anions considered in this study: I^−^, Br^−^, F^−^, thiocyanate^‐^ (SCN^−^), or methanesulfonate^−^ (MS^−^), as described before (See Solutions in the Methods section). Figure 4 shows two groups of Figures. The upper one belongs to recordings made to study the selectivity of (ICl‐G1), whereas the lower belongs to ICl‐G2. Figure A of each group shows representative traces of currents generated by the ramp function for each of the anions considered in the study. Figure B shows the average values of the reversal potential in each case, while Figure C shows the relative permeability values calculated from the change of reversal potential, according to the Goldman–Hodgkin–Katz equation (described in Methods). These results show that the outward component of currents has a different sequence of selectivity than the inward one, being that of the first SCN^−^>I^−^>Br^−^>Cl^−^>F^−^>MS^−^, while that of the second is Cl^−^>Br^−^>I>SCN^−^>F^−^>MS^−^.

### Hypoosmotic challenge reveals third type of currents (ICl‐G3) and produces changes in the inwardly rectifying type (ICl‐G1)

**Figure 5:**
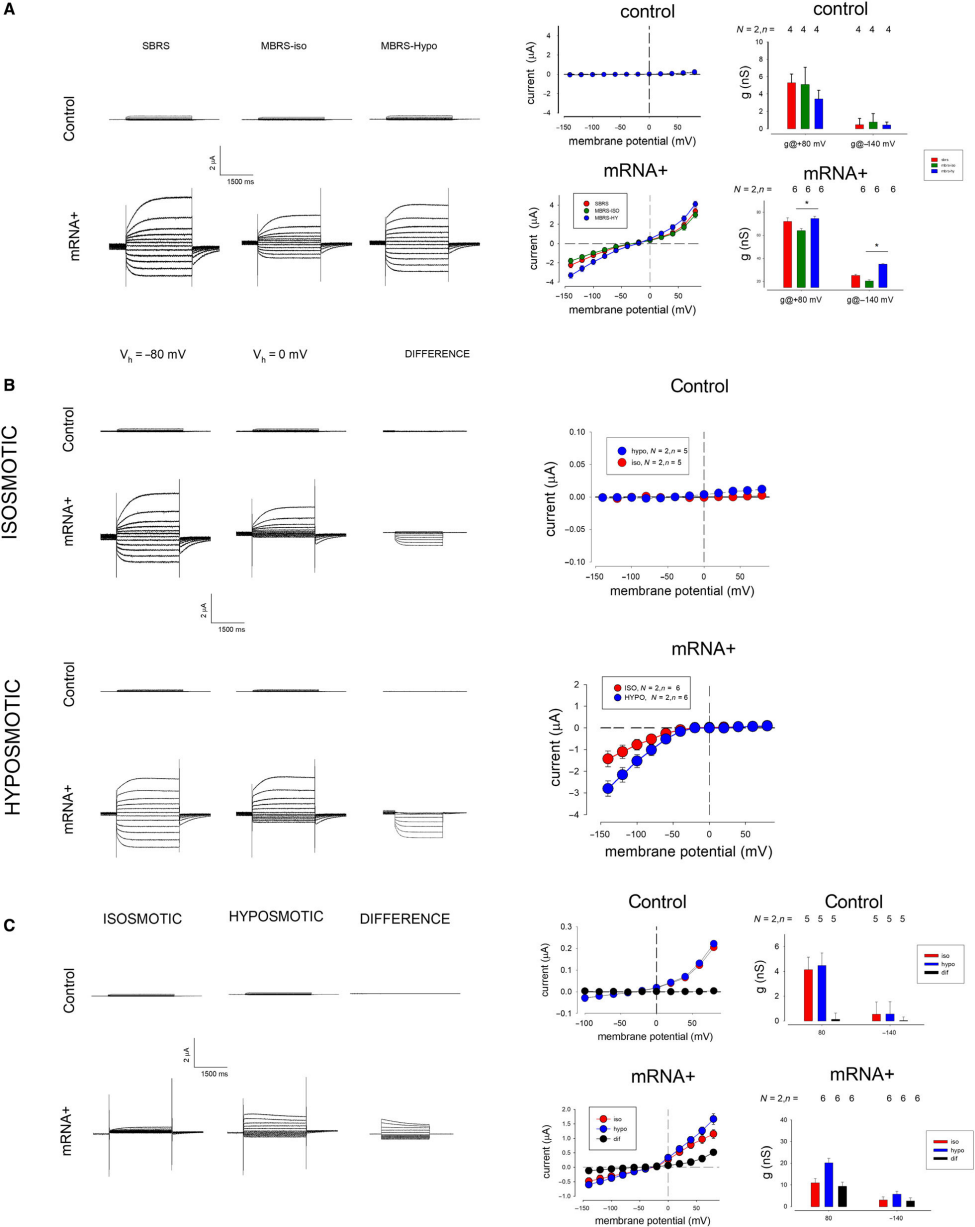
Hypoosmotic challenge enhances ICl‐G1 and reveals a third type of current (ICl‐G3).(A) 1. Series of representative currents, obtained from an oocyte, 3 days after injection with *Giardia*′s mRNA, under the same protocol of stimulation, while bathed with a normal solution SBRS (left), then with a modified, isosmotic solution MBRS‐ISO (middle), then with a modified hypoosmotic solution (right). 2. Time course of the response to hypoosmotic challenge of mRNA‐injected oocytes. Alternate test pulses to +80 and −140 mV were given to a mRNA‐injected oocyte every 30 sec during 4 min. 3. IV relationships of currents obtained from control (upper) and mRNA‐injected (lower) oocytes while bathed with either SBRS, MBRS‐ISO, or MBRS‐HO. 4. Statistical comparison of the average slope conductance at −140 or +80 mV of control (upper) and mRNA‐injected (lower) oocytes while bathed with the three distinct media. Pairwise comparison (*t*‐test) indicates statistically significant difference (*P* < 0.05) for values before and after hypoosmotic challenge at −140 and +80 mV of mRNA‐injected but not of control oocytes. (B) 1. Representative series of currents of control (upper) and mRNA‐injected (lower) oocytes, bathed with an isoosmotic (upper set) or a hypoosmotic (lower set) solution, oocytes were stimulated with a prepulse of −80 mV (left) and 0 mV (middle) to obtain a difference, corresponding to ICl‐G1. It can be observed that hypoosmotic challenge enhances the magnitude of the difference (ICl‐G1) currents of mRNA‐injected but not from control oocytes. 2. IV relationship of the difference (ICl‐G1) current, before (red circles) and after (blue circles) of control (upper) and mRNA‐injected oocytes. (C) 1. Representative series of currents obtained from a control (upper row) and a mRNA‐injected (lower row) oocyte, in both cases oocytes were injected with BAPTA to inhibit outward (ICL‐G2) currents, and with a prepulse of 0 mV to minimize ICl‐G1 currents. A new type of current (ICl‐G3) is revealed by substracting currents under hypoosmotic and isoosmotic conditions recorded from mRNA‐injected oocytes, whereas those from control oocytes observed no difference currents. 2. IV relationships from recordings of control (upper) and mRNA‐injected (lower) oocytes under the distinct experimental conditions described above, the number of cases is the same as in figure (C.3). 3. Comparison of the slope conductances at +80 and −14 mV of control (upper) and mRNA‐injected (lower) oocytes under isosmotic and hypoosmotic conditions, as well as their difference. Statistical analysis (*t*‐test) indicates that the slope conductance at +80 mV of mRNA‐injected oocytes is significantly distinct from 0 (*P* < 0.01)

We designed a series of experimental assays to determine whether hypoosmolarity affects ICl‐G currents. For this purpose, we replaced the normal SRBS with (MRBS‐ISO) a solution in which we replaced 36 mmol/L NaCl by 72 mmol/L mannitol, in order to be able to play with osmolarity without affecting the ionic composition (see Table 1). Figure 5A.1 shows several series of representative current recordings, those in the upper row were recorded from a control (uninjected) oocyte, whereas those in the lower one were obtained from an oocyte injected with *Giardia*′s mRNA 3 days before recording. All these recordings were produced with the same protocol as in Figure 1. The left column shows recordings from oocytes bathed with SRBS, the middle one shows recordings of the same oocytes while bathed with MRBS‐ISO and the left column with MRBS‐HO (hypoosmolar). Figure 5A.2 shows the time course of the response to the hypoosmotic challenge of oocytes injected with mRNA. Figure 5A.3 shows the IV relationships obtained in the three distinct bathing solutions from control (upper) and injected (lower) oocytes. Figure 5A.4 compares the slope conductance at −140 and +80 mV of control (upper) and injected (lower) oocytes.

In both cases (control and mRNA‐injected oocytes), the change of bathing media, from SRBS to HBRS‐ISO produce a reduction in the magnitude of currents, although their kinetics remains the same. The hypoosmolar challenge, made by changing the bathing media to MBRS‐HO, produces enhancement of currents, both in control and in mRNA‐injected oocytes. Currents from control oocytes increase their magnitude in the depolarizing range and do not change their kinetics. In contrast, currents from injected oocytes increase their magnitude, both in the hyperpolarizing as in the depolarizing range. Also, it can be observed a change in the kinetics of outward currents, which became slower. These results lead us to suspect that the hypoosmotic challenge is directly affecting one or both of the two components already described (ICl‐G1 or ICl‐G2), or that it is inducing the expression of a third component.

To assess the possibility that ICl‐G1 is affected by hypoosmolarity, we made a procedure to dissect it out, by subtracting two series of ion current recordings, made consecutively in the same oocyte, the first with a prepulse of 0 mV and the second with a prepulse of −80 mV. Figure 5B.1 shows two sets of representative currents made to measure ICl‐G1 under isosmotic (upper) and hypoosmotic (lower) conditions. In each of these sets, the results of a control oocyte (top) with an injected one are compared (below).

It is observed that in the case of control oocytes this procedure of substraction does not produce a noticeable difference, whereas in injected oocytes, substraction reveals incoming currents (as previously described). Figure 5B.2 compares the IV relationship under isosmotic (red circles) and hypoosmotic conditions of control (upper) and mRNA‐injected oocytes. It is also clear that hypoosmotic challenge enhances the currents disclosed after substraction, which are attributable to ICl‐G1.

On the other hand, to determine whether hypoosmotic challenge induces the expression of a third component, we seek to minimize the magnitude of ICl‐G1 and ICl‐G2, therefore, we made recordings of ion currents from oocytes that were previously injected with BAPTA, to suppress ICl‐G2, and held the prepulse voltage to 0 mV, to reduce the contribution of ICl‐G1. Figure 5C.1 shows series of currents recorded from oocytes, control (upper), and mRNA‐injected (lower) treated under those experimental conditions, before (left) and 5 min (middle) after hypoosmotic challenge. It is shown that anion currents from control oocytes are not modified after hypoosmotic challenge, as substraction reveals no conspicuous currents, while the same procedure applied to mRNA‐injected oocytes reveals currents whose shape is entirely different to those previously described (ICl‐G1 and ICl‐G2). These currents also exhibit outward rectification, but, unlike IC1‐G2, they do not activate gradually over time, but have a maximum magnitude at the time of voltage change. Also, they display inactivation in response to voltage pulses above +60 mV. Figure 5C.2 shows the IV relationship before and after the hypoosmotic challenge, from control (above) and mRNA‐injected (below) oocytes; Figure 5C.3 compares the slope conductance of currents (before, after hypoosmotic challenge and difference) at −140 and +80 mV. Statistical analysis (*t*‐test) indicates that the slope conductance of difference current is distinct from zero (*P* < 0.01). These results, therefore, lead us to assert that injection of *Giardia*′s mRNA induces expression of a third type of anion currents (ICl‐G3), which are revealed only after hypoosmotic challenge.

### Pharmacological properties of chloride currents expressed in oocytes by injection of *Giardia* mRNA

We assayed the effect of several chloride channel blockers on the three distinct types of current (ICl‐G1, ICl‐G2, and ICl‐G3) described above. The concentrations tested were similar to those probed in other works, described in the literature (Ackerman et al. 1994; White and Aylwin 1990; Furukawa et al. 1998; Schmieder et al. 1998; Ponce et al. 2008).

**Figure 6:**
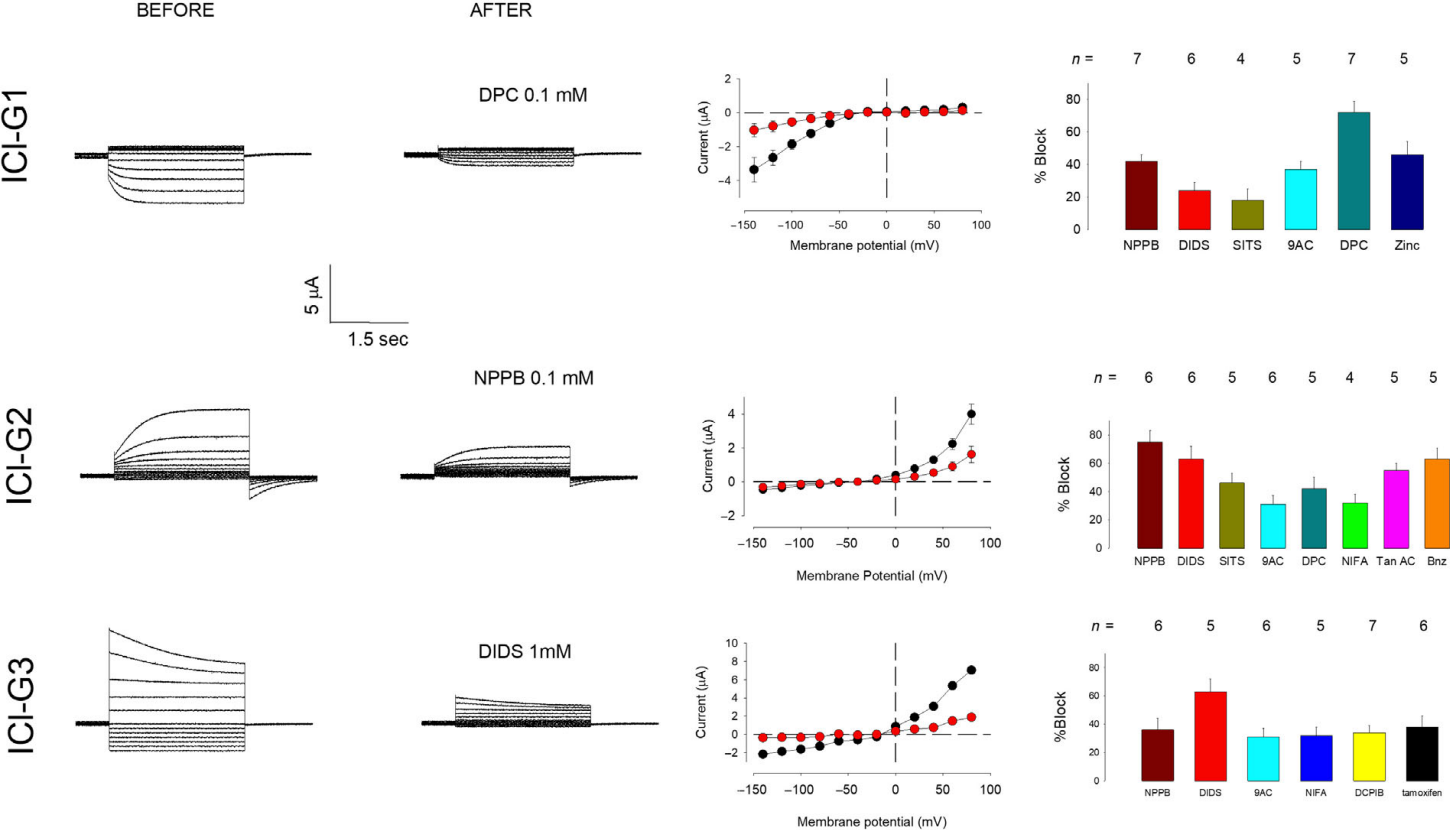
Pharmacological properties of chloride currents induced by injection of mRNA of *Giardia*. The properties of the exogenous currents (IClG1‐G3) are shown in three rows and four columns. The first two columns (from left to right) show a representative example, before and after the addition of the blocker indicated in the second column, the third column shows the IV relationship before (black circles) and after (red circles) the addition of the blocker. The fourth column shows the effect of distinct chloride channel blockers on each type of currents. The results are expressed as % blocking, under the experimental conditions described in Results.

For the first type of current (ICl‐G1), we assayed DIDS (1 mmol/L), NPPB (0.1 mmol/L), 9‐Anthracene (9AC, 1 mmol/L), and Zinc (40 *μ*mol/L). To measure the blocking effect of each compound on (ICl‐G1), we recorded ion currents. First, after a prepulse of −80 mV for 5 sec, we recorded the current produced by a test pulse at −140 mV, then made a second trial with a prepulse of 0 mV and annotated the difference in amplitude of those two trials. We repeated this procedure after 5 min of addition of the testing drug to the bathing media. To calculate the percentage of inhibition, the difference in the amplitude of the current, before and after addition of the drug, was divided by the value of amplitude before addition of the drug, and this value is referred to as a percentage. Figure 6 shows in the first row, from left to right: (1) A representative series of ICl‐G1 currents before and (2) after addition of DPC 0.1 mmol/L to the media; (3) The IV relationship before and after addition of DPC, and (4) a bar series of the blocking effects of drugs tested for ICl‐G1. NPPB (0.1 mmol/L) blocked 42 ± 4% (*n* = 5), DIDS (1 mmol/L) 24 ± 5% (*n* = 5), SITS (1 mmol/L) 18 ± 7% (*n* = 4), 9AC(1 mmol/L) 37 ± 5% (*n* = 5), DPC(0.1 mmol/L) 72 ± 7% (*n* = 4), Zinc(0.05 mmol/L) 46 ± 8% (*n* = 4).

For the second type of currents (ICl‐G2), we assayed the effect of the general chloride channel blockers: NPPB, DIDS, SITS, 9AC, DPC, and Niflumic Acid (NIFA). Also, we tested the blocking effect of tannic acid and benzbromarone, which had been described to block TMEM16 A/B calcium‐activated chloride channels (Huang et al. 2012).

To measure the effect of each blocker, we recorded the current produced by a testing pulse of +80 mV after a prepulse of 0 mV from oocytes injected with mRNA, bathed with SRBS, before and 5 min after addition of the testing drug. From those two measurements, we calculated the percent blocking as explained before. The results are shown in the middle row of Figure 6. NPPB (0.1 mmol/L) blocked 75 ± 8% (*n* = 5), DIDS (1 mmol/L) 63 ± 9% (*n* = 4), SITS (1 mmol/L) 46 ± 7% (*n* = 5), 9AC(1 mmol/L) 31 ± 6%(*n* = 4), DPC(0.1 mmol/L) 42 ± 8% (*n* = 4), NIFA(0.1 mmol/L) 32 ± 6% (*n* = 4), tannic acid (0.1 mmol/L) 55 ± 5% (*n* = 4), and benzbromarone (0.01 mmol/L) 63 ± 8% (*n* = 5).

For the third type of chloride currents (ICl‐G3), we tested the chloride channel blockers NPPB, DIDS, 9AC, and NIFA. Also, given that these currents show properties similar to ICl‐swell, we tested the effect of DCPIB and tamoxifen, which have been described to block these type of currents (Okada 1997). To measure the blocking effect of each compound, we made recordings of currents from oocytes that had been injected with mRNA 2 days before and injected with BAPTA 5 min before, to suppress ICl‐G2. The oocyte was hypoosmotically challenged 5 min before recording. ICl‐G3 Currents were induced by a prepulse of 0 mV for 2 sec (To minimize ICl‐G1, followed by a test pulse of +80 mV). This was repeated after 5 min of addition of the testing drug to the bathing media. The percent blocking value was calculated from those two measurements. The results are shown in the lower row of Figure 6. NPPB (0.1 mmol/L) blocked 36 ± 8% (*n* = 5), DIDS (1 mmol/L) 63 ± 8% (*n* = 4), 9AC (1 mmol/L) 31 ± 9% (*n* = 3), NIFA (0.1 mmol/L) 32 ± 6% (*n* = 4), tamoxifen (0.1 mmol/L) 38 ± 8% (*n* = 5), and DCPIB (0.01 mmol/L) 34 ± 5% (*n *= 4).

## Discussion

*Giardia lamblia* is one of the most common parasites found in the human intestinal tract which causes giardiasis, an acute or chronic infection worldwide. Despite its importance in terms of health, little is known about its cellular physiology, especially about the diversity of ion channels that this protozoan expresses.

In the present study, we show that mRNA injection, isolated from trophozoites of *Giardia*, induces the expression of chloride currents in *Xenopus laevis* oocytes (ICl‐G). The biophysical and pharmacological properties of these currents are different to those expressed by noninjected oocytes and disappear when degraded mRNA is injected instead. These results indicate, therefore, that (ICl‐G) are the result of the expression of *Giardia* chloride channels in the plasma membrane of *Xenopus oocytes*. Our results also indicate that ICl‐G is composed of two types of currents (ICl‐G1 and ICl‐G2).

The biophysical and pharmacological properties of ICL‐G1 are somewhat comparable to those exhibited by currents generated by ClC2 channels in other species, including inward rectification, activation by hyperpolarizing voltages, and cell swelling. Also, the halide anion permeability sequence of ICl‐G1 (Cl^−^>Br^−^>I^−^>SCN^−^>F^−^>MS^−^), is comparable to that reported for ClC2 (Cl^−^≥Br^−^≫I^−^≥F^−^) (Fahlke 2001). Likewise, ICl‐G1 and mammalian IClC2 are comparable in most of their pharmacological properties; It has been reported that ClC‐2 currents are inhibited by NPPB (0.5 mmol/L), DPC (1 mmol/L), 9AC (1 mmol/L), and Zinc (40 *μ*mol/L), but are largely unaffected by SITS and DIDS (1 mmol/L) (Furukawa et al. 1998; Duan et al. 2000; Bi et al. 2013). ICl‐G1 are also inhibited by NPPB, DPC, 9AC, and Zinc but, unlike IClC2, ICl‐G1 were weakly, although significantly, inhibited by DIDS and SITS. These results agree with those reported by Moreno‐Galindo and co‐workers, whom, making whole‐cell clamp assays, described a conductance similar to ClC‐2 in trophozoites of *Giardia* (Moreno‐Galindo et al. 2014).

ICl‐G2, on the other hand, is a type of chloride current that depends of intracellular calcium (ICl,Ca) because, as we showed, ICl‐G2 currents were abolished after chelating the intracellular content of free calcium, by injection of BAPTA into oocytes previously injected with mRNA of *Giardia*, however ICl‐G2 currents are restituted after incubation with ionomycin, which premeabilizes the plasma membrane, and addition of calcium to the bathing media. Besides its dependence of intracellular calcium, the permeability sequence of ICl‐G2 (SCN^−^>I^−^>Br^−^>Cl^−^>F^−^>MS^−^) compares to that of CaCC channels: SCN^−^> I^−^ > NO_3_^−^ > Br^−^ > Cl^−^ > F^−^ (Kamaleddin 2018). The pharmacological profile is also similar; As shown above, ICl‐G2 are blocked by NPPB, DIDS, SITS, 9AC, and DPC, whereas CaCC are described to be blocked by niflumic acid, NPPB, 9AC, and DIDS. (Sanders et al. 2014). Additionally, we found that ICl‐G2 are blocked by tannic acid and benzbromarone. These two compounds have been reported to inhibit currents generated by expression of TMEM16A/B channels (Huang et al. 2012), which have been described as the molecular entities that produce CaCC channels (Schroeder et al. 2008; Pifferi et al. 2009; Pang et al. 2014). Our results, therefore suggest that these currents are produced by channels, somehow related to TMEM16A/B. Interestingly, Tannic acid, a compound that is found in red wines and green tea (Namkung et al. 2010), has been proposed as therapy for diarrheal infections (Das et al. 2018). Therefore, these results indicate that ICl‐G2 are a type of calcium‐dependent chloride currents (ICl,Ca) produced by chloride channels that depend of intracellular calcium (CaCC).

The third type of currents (ICl‐G3) is revealed only after hypoosmotic challenge to oocytes injected with mRNA of *Giardia*. The biophysical properties of ICl‐G3 are somewhat comparable to those described for ICl‐swell, a type of chloride currents produced by VRAC, also known as volume‐sensitive outwardly rectifying (VSOR) anion channel (Nilius et al. 1994; Pedersen et al. 2015). In addition to its role in cell volume homeostasis, VRAC are involved in numerous other physiological and pathophysiological processes, including cancer, ischemic brain edema, cell motility, proliferation, angiogenesis, and programmed cell death (Pedersen et al. 2016).

Some of the biophysical properties, that have been described for ICl‐swell, and that are also exhibited by ICl‐G3 are: (1) Activation by osmotic cell swelling (2) Outward rectification, and (3) Inactivation at large positive potentials (Akita and Okada 2014; Hoffmann et al. 2014). Their pharmacological properties are likewise comparable: ICl‐swell currents have been reported to be blocked by conventional nonselective anion channel blockers, DIDS, SITS, NPPB, and niflumic acid (Okada 2006). In some cell types, Tamoxifen, a competitive antagonist of estrogen receptors, has been reported to block VRAC channels (Okada 1997). DCPIB has been described as the most selective inhibitor of VRAC channels currently available (Decher et al. 2001; Akita and Okada 2014). As shown, ICl‐G3 was inhibited by NPPB, DIDS, 9AC, niflumic acid, tamoxifen, and DCPIB. It is important to note, however, that DCPIB did not inhibit the currents in the way that has been demonstrated in other works. For example, it has been described that DCPIB (10 *μ*mol/L) inhibits in 90% the ICl‐swell currents of calf bovine pulmonary artery endothelial cells (Decher et al. 2001). In our case, we observed that this concentration produced an inhibition of only 33%, this could indicate important structural or functional differences between the chloride channels that produce such currents in mammals as in *Giardia*.

In conclusion, our results indicate that trophozoites of *Giardia* express at least three types of chlorine channels, with characteristics similar to those described in other organisms to channels type ClC2, CaCC, and VRAC. The physiological roles of these channels in *Giardia*, is likely to be similar to those described in other organisms, including regulation of the membrane potential, volume regulation, recognition of the environment. Although these results may not be surprising, since such chloride channels are ubiquitous in all cell types, it is worth considering that the experimental approach described here could be used to find new chemical compounds that, by selectively inhibiting the chloride channels of *Giardia*, could also function as therapeutic agents against giardiasis.

## Conflict of Interest

None declared.
